# Functional modification of adipocytes by grape seed extract impairs their pro-tumorigenic signaling on colon cancer stem cells and the daughter cancer cells

**DOI:** 10.18632/oncotarget.2467

**Published:** 2014-10-10

**Authors:** Sushil Kumar, Dileep Kumar, Komal Raina, Rajesh Agarwal, Chapla Agarwal

**Affiliations:** ^1^ Department of Pharmaceutical Sciences, Skaggs School of Pharmacy and Pharmaceutical Sciences, University of Colorado Anschutz Medical Campus, Aurora, CO 80045, USA; ^2^ University of Colorado Cancer Center, University of Colorado Anschutz Medical Campus, Aurora, CO 80045, USA

**Keywords:** colorectal cancer, obesity, chemoprevention, phytochemicals, adipocytes

## Abstract

With global rise in obesity, it is imperative that we identify obesity-driven factors that increase growth and progression of colorectal cancer (CRC), and also discover and develop agents with anti-CRC efficacy under obese conditions. Here in, we investigated grape seed extract (GSE), a well-defined agent with both preventive and anti-CRC efficacy, for its potential to impair pro-tumorigenic signaling of adipocytes on CRC/colon cancer stem cells (CSCs) and associated molecular mechanisms, to control CRC under obese conditions. GSE treatment significantly decreased the growth and invasion promoting effects of both mouse and human adipocytes on CRC cells. Moreover, GSE exerted a direct inhibitory effect, as well as it strongly reduced the growth promoting signals of adipocytes, on colon CSCs. These GSE effects were associated with a decrease in both mRNA and protein levels of various CSC-associated molecules. Notably, GSE effects on adipocytes were not due to changes in lipid content, but by inducing the ‘browning’ of adipocytes as evidenced by an increase in *UCP-1* mRNA level and mitochondriogenesis. Together, these findings, for the first time, suggest the ability of GSE to induce ‘brown remodeling’ of white adipocytes, which causes functional modification of adipocytes thus impairing their pro-tumorigenic signals on colon CSCs/CRC cells.

## INTRODUCTION

Colorectal cancer (CRC), the third leading cause of cancer incidence in US (both genders) [1], has additional increased risks combined with obesity, type II diabetes and/or inflammatory bowel disease [2–7]. Furthermore, obesity is associated with increased CRC incidence and mortality [8]. The systemic obesity-associated metabolic aberrations, such as dyslipidemia, hyperglycemia, insulin resistance and increased inflammatory signals, have the potential to affect CRC growth [3–7]. Since colon lies in close anatomical vicinity of fat depots, peritoneal secretions from obese dysfunctional adipose tissue may serve as the most significant source of pro-tumorigenic and inflammatory factors to drive an enhanced growth and progression of CRC under obese conditions [9–11]. Clearly, worldwide obesity epidemic poses a major challenge to CRC [8], and thus underscores the need for new approaches to identify and define the critical factors which drive the interactions between adipocytes signaling (systemic and peritoneal) and CRC cell as well as colon cancer stem cells (CSCs); importantly, CSCs are now recognized as the main cause for the growth and progression of most epithelial cancers, including CRC [12–18]. Also notably, compared to lean condition, obesity is manifested by an increase in white adipose tissue (WAT) mass; a hypoxic WAT condition where hypertrophied adipocytes fail to store excess triglycerides leading to ectopic lipid deposition and metabolic disorders [9–11]. This WAT dysfunction and hypoxic environment are associated with increased infiltration of immune cells into the adipose tissue causing a low grade inflammation [4, 9–11]. This obesity-induced inflammation, with increased secretion of pro-inflammatory factors from the adipocytes and inflammatory cells [9–11], can promote the expansion of colon cancer stem cells (CSCs) pool resulting in an enhanced CRC growth and progression under obese condition. Thus, an agent with both anti-obesogenic and anti-CRC activities would be ideal to impair different facets of adipocyte-CRC interaction resulting in CRC prevention and control under obese conditions. Notably, significant efforts have been made in last two decades evaluating the chemopreventive potential of a wide range of agents against CRC, but mostly in non-obese conditions [19–21].

Certainly, a critical gap exists in our knowledge regarding the efficacy of these agents against CRC under obese conditions. Accordingly, in this study, we investigated the potential of grape seed extract (GSE) to inhibit adipocytes-driven pro-tumorigenic signaling on both human CRC and colon CSCs in the context of CRC prevention and control under obese conditions. GSE, a well-defined chemical entity containing procyanidins [20, 22, 23], has shown strong preventive and therapeutic efficacy in various CRC *in vitro* and rodent models [19, 20, 23–32] including azoxymethane (AOM)-induced aberrant crypt foci in Fisher 344 rats [31], AOM-induced colon tumorigenesis in A/J mice [26], spontaneous intestinal tumors in APC^min/+^ mice [32] and CRC cell xenografts in nude mice [29], together with a decrease in proliferative and an increase in apoptotic indices [26, 29, 31, 32]. Also importantly, a series of animal studies have shown that GSE partially alleviates the high fat diet-induced obesity, decreases the weight of fat pads, and suppresses the body weight increase together with an improvement in associated metabolic abnormalities [33–36]. Several of these effects of GSE were attributed to interference in dietary fat absorption/accumulation due to its inhibitory effect on fat metabolizing enzymes (lipases), an increase in lipolytic genes, and a decrease in lipogenic genes [33–38]. Furthermore, during high fat diet-induced hyperlipidemia, GSE has been shown to normalize triglyceride and total cholesterol levels in serum and liver with a concomitant increase in high density cholesterol [38, 39]. GSE is also reported to prevent ectopic fat deposition and associated lipotoxicity in rodents [38, 40]. Taken together, these published studies clearly suggest that GSE possesses both anti-CRC and anti-obesogenic activities, and thus it is a novel agent to be studied in detail for its efficacy against CRC under obese conditions. Thus, herein, for the first time, we studied the impact of adipocyte secretions from mouse, non-diseased human, and diabetic human variants, on the CRC/CSC cell populations, specifically defining the role of adipocytes on growth and invasive potential of human CRC and then determining whether GSE intervention could impair these cancer promoting signals of adipocytes.

## RESULTS

### GSE does not interfere with adipocyte differentiation and lipid accumulation in mature adipocytes

**Figure 1:**
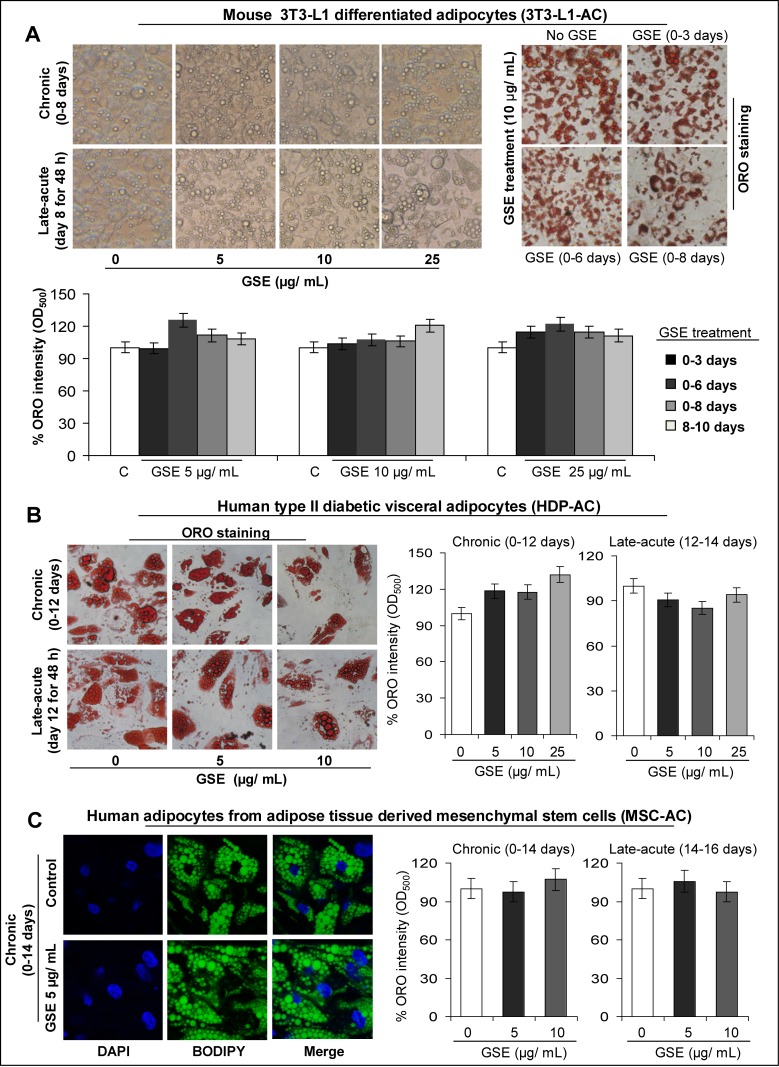
Effect of GSE on adipocyte differentiation and lipid accumulation in mature mouse and human adipocytes **(A)** GSE (selected optimum concentration) does not have any effect on adipocytes maturation (chronic treatment) or mature adipocytes (acute treatment) in 3T3-L1 adipocytes. Representative photomicrographs of, *A-left panel)* phase contrast microscopy; and *A-right panel)* Oil Red-O (ORO, X 100 magnification) staining of lipids in the absence and presence of GSE treatment are shown. *A-bottom panel)* Percent ORO staining intensity (O.D.500) relative to control is shown after chronic and acute treatments with GSE. Staining was done after adipocyte maturation. **(B)** GSE (selected optimum concentration) does not have any effect on adipocytes maturation (chronic treatment) or mature adipocytes (acute treatment) in human type II diabetic visceral adipocytes (HDP-AC). *B-left panel)* Representative photomicrographs of Oil Red-O (ORO, X 400 magnification) staining of lipids in the absence and presence of GSE treatment are shown. *B-right panel)* Percent ORO staining intensity (O.D.500) relative to control is shown after chronic and acute treatments with GSE. Staining was done after adipocyte maturation. **(C)** GSE (selected optimum concentration) does not have any effect on adipocytes maturation (chronic treatment) or mature adipocytes (late-acute treatment) in human adipocytes from adipose tissue derived mesenchymal stem cells [MSC-AC]. *C-left panel)* Representative photomicrographs of BODIPY-493/503 (X 600 magnification) staining of lipids in the absence and presence of GSE (5μg/mL) chronic treatment are shown. *C-right panel)* Percent ORO staining intensity (O.D.500) relative to control is shown after chronic and acute treatments with GSE. Staining was done after adipocyte maturation. Columns, mean values; error bars, SEM.

First, we carried out chronic and late-acute treatment protocols (Supplementary Figure 1) with different doses of GSE to determine its effect on: i) adipocyte differentiation and ii) lipid accumulation in mature adipocytes, in a panel of mouse/human adipocytes [mouse 3T3-L1 differentiated adipocytes, human type II diabetic visceral adipocytes, and human adipocytes from adipose tissue-derived mesenchymal stem cells; hereafter, these adipocytes have been abbreviated as 3T3-L1-AC, HDP-AC, and MSC-AC, respectively, in all the Figures]. For chronic GSE treatment, post-confluent mouse 3T3-L1 fibroblasts, human type II diabetic visceral pre-adipocytes, and human adipose tissue-derived mesenchymal stem cells were exposed to GSE doses (2.5–25 μg/mL in DMSO) for short and long intervals during the process of adipogenesis to identify non-toxic GSE doses that cause adipocyte functional modulation without affecting their maturation (Supplementary Figure 1). For late-acute GSE treatment, mature adipocytes were treated with GSE for 48 h (Supplementary Figure 1). In both protocols, GSE effect on adipocyte maturation was determined by Oil Red-O (ORO) or BODIPY-493/503 staining of lipids (Figure 1).

As shown in Figure 1A, mouse 3T3-L1 fibroblasts (pre-adipocytes) treated chronically with 5–25 μg/mL GSE for variable durations (0–3 days, 0–6 days, and 0–8 days) or mouse 3T3-L1 differentiated adipocytes exposed to late-acute GSE treatment (day 8 for 48h) did not show any effect on lipid content as determined by phase contrast images depicting confluent mouse 3T3-L1 differentiated adipocytes with abundant translucent oily droplets within the cells (Figure 1A-*left panel*), ORO staining of lipids (red droplets in Figure 1A-*right panel*), and percent ORO staining intensity (O.D.500) relative to respective untreated controls (Figure 1A-*bottom panel*). Similarly, human type II diabetic visceral pre-adipocytes treated chronically with 5–25 μg/mL GSE for 0–12 days (Figure 1B-*right panel*) or matured human type II diabetic visceral adipocytes exposed to late acute GSE treatment (day 12 for 48 h, Figure 1B-*right panel*) did not show any significant changes in ORO staining intensity. Representative ORO staining of lipids in these cells is shown in Figure 1B-*left panel*. Chronic GSE treatment (0–14 days) of human adipose tissue-derived mesenchymal stem cells (Figure 1C-*right panel*) or late-acute GSE treatment (day 14 for 48h) of mature human adipocytes from adipose tissue-derived mesenchymal stem cells (Figure 1C-*right panel*) also did not affect their percent ORO staining intensity. Representative BODIPY-493/503 staining of lipids depicting abundant lipid content in control and GSE-treated cells is shown in Figure 1C-*left panel*.

Overall, based on ORO staining of lipids and percent ORO staining intensity (Figure 1A-C), the optimal GSE concentrations, selected for the future experiments that did not have any effect on adipocytes maturation or mature adipocytes (based on no changes in lipid content), were 10–25 μg/mL for mouse 3T3-L1 differentiated adipocytes, 10 μg/mL for human type II diabetic visceral adipocytes, and 5 μg/mL for human adipocytes from adipose tissue-derived mesenchymal stem cells. Due to the complex process of adipogenesis under *in vitro* experimental conditions, which necessarily requires the cells to be over confluent for adipocyte maturation to ‘set in’, it was not possible to measure lipid content with respect to adipocyte cell number. Since GSE at the selected optimum doses did not affect the viability of either the pre-adipocytes or the mesenchymal stem cells as determined by Trypan blue exclusion assay (data not shown), it was evident that the correction for cell number was not a limiting factor in the adipogenesis experiments carried out in the presence of GSE. Furthermore, since there was no change in the lipid content, it indicates that the selected optimum non-toxic GSE doses do not interfere in differentiation of pre-adipocytes and mesenchymal stem cells to mature adipocytes and that these GSE doses also do not affect the lipid content of mature adipocytes.

### GSE interferes in adipocyte functional signaling by inducing ‘browning’ of adipocytes

**Figure 2:**
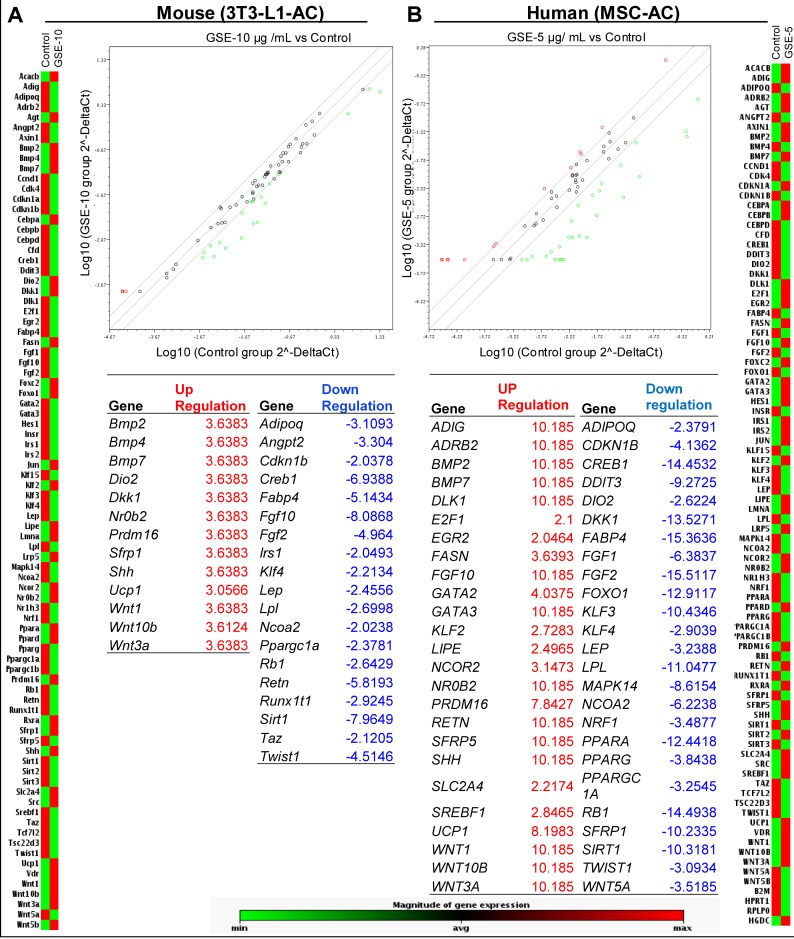
Effect of GSE on mRNA levels of adipogenesis-associated genes in mouse and human adipocytes Representative heat map showing relative changes in genes estimated by RT^2^qPCR Profiler ^TM^ PCR Array in, **(A)** mouse 3T3-L1 differentiated Adipocytes (3T3 L1-AC), and **(B)** Human adipocytes from adipose tissue derived mesenchymal stem cells (MSC-AC). Total RNA was isolated from mouse 3T3-L1-AC after chronic GSE (10 μg/mL, 0–8 days) treatment, and from MSC-AC after chronic GSE (5 μg/mL, 0–14 days) treatment.

Next, we examined whether GSE (at the selected optimum doses defined above) modulates the expression of mRNAs and/or proteins which are essential for the adipocyte functional signaling. We used mouse/human adipogenesis RT^2^ qPCR Array (Qiagen) to analyze the expression of genes associated with adipogenesis in the mouse 3T3-L1 differentiated adipocytes and human adipocytes from adipose tissue-derived mesenchymal stem cells which were exposed to chronic GSE treatment (Figure 2). Representative heat maps showing the relative changes in the genes estimated by RT^2^ qPCR indicated that GSE causes an alteration in the expression of various adipogenesis-related genes in both mouse (Figure 2A) and human (Figure 2B) adipocytes.

**Figure 3:**
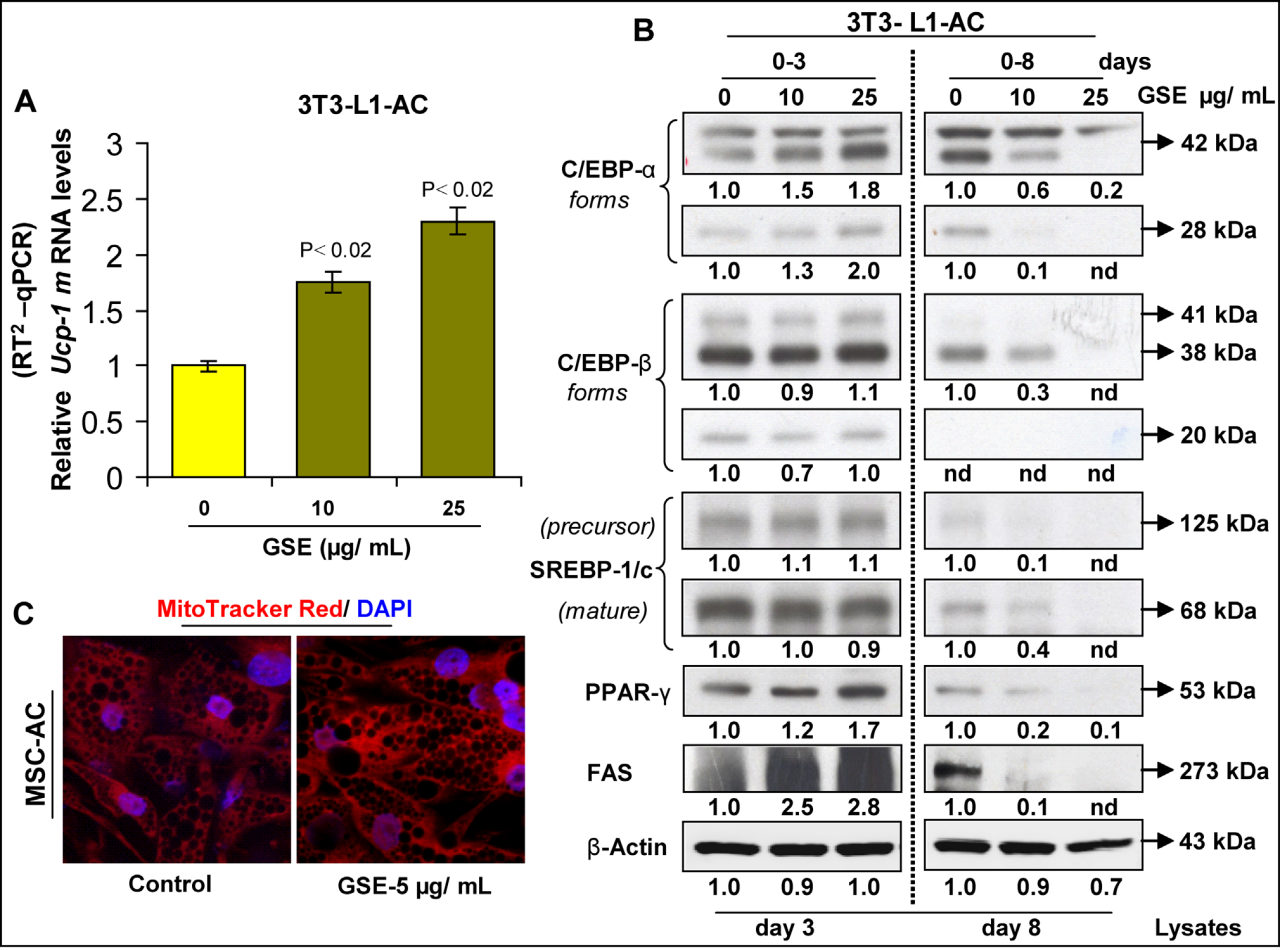
Effect of GSE on the browning of adipocytes GSE increases **(A)** mRNA levels of *Ucp-1* in mouse 3T3-L1differentiated adipocytes (3T3-L1-AC) as analyzed by RT^2^qPCR after chronic GSE treatment. **(B)** Western blot analysis of adipogenesis-related proteins during different stages of 3T3-L1 adipocyte differentiation by GSE; β-actin was used as loading control after stripping and re-probing the blots. Relative densitometric values compared to respective controls are shown below each blot (nd, not detectable bands). **(C)** GSE increases mitochondriogenesis in human adipocytes from adipose tissue derived mesenchymal stem cells (MSC-AC) after chronic treatment [GSE dose: 5ug/mL (0-14 days)]. Representative images (X 600 magnification) show MitoTracker Red stained mitochondria and DAPI-blue stained nuclei, in the absence and presence of GSE. Columns, mean values; error bars, SEM.

Notably, in mouse 3T3-L1 differentiated adipocytes (Figure 2A), GSE caused an increase (3 folds) in the expression of pro-brown adipose tissue (BAT) genes *Ucp-1, Prdm16, Bmp7 and Dio2*,while it decreased the expression of anti-BAT *Twist1* and also increased anti-BAT *Wnt10b and Nr0b2 gene* levels. Also, the levels of pro-WAT genes *Fgf10 and Klf4* were decreased strongly by GSE, but there was also an increase in *Bmp2 and Bmp4*; however, no effect on anti-WAT and pro-adipogenesis gene levels was observed by GSE. In these cells, the gene levels of adipokines, such as *Adipoq*, *Lep* and *Retn* were also decreased by GSE treatment. Consistent with its effect in mouse 3T3-L1 differentiated adipocytes, GSE also increased the level of pro-BAT *UCP-1* gene by ~8 folds in human adipocytes from adipose tissue-derived mesenchymal stem cells (Figure 2B); other pro-BAT genes that were significantly increased (~8-10 folds) by GSE were *PRDM16* and *BMP7*. However, not all pro-BAT genes were increased by GSE in these cells; some pro-BAT genes such as *DIO2, CREB1, PPAR-α* and *PGC1-α* were decreased. The levels of pro-WAT gene *KLF4* was decreased by GSE in these cells, but the levels of *BMP2, EGR2, FGF10 and SREBF1* were increased. Furthermore, while anti-WAT genes such as *GATA2, GATA3 and KLF2* were increased, the anti WAT *KLF3* gene level was decreased by GSE. The pro-adipogenesis genes that were significantly decreased by GSE were *DKK1, FABP4, FGF1* and *FGF2*, while the levels of genes, such as *E2F1, FASN, SFRP5* and *SLC2A4* were increased. The gene levels of adipokines such as *ADIPOQ* and *LEP* were decreased by GSE treatment but the levels of *ADIG* and *RETN* were significantly increased. Furthermore, the levels of lipoprotein lipase (LIPE) gene were also significantly decreased by GSE treatment in both mouse 3T3-L1 differentiated adipocytes and human adipocytes from adipose tissue-derived mesenchymal stem cells. A comprehensive analysis of all these results clearly suggests that the alterations in most of the genes were in favor of adipocyte browning. Since the expression of pro-browning indicator gene UCP-1 was strongly increased in both cell lines, its increased mRNA expression was further confirmed by using specific primers for mouse *Ucp-1* by RT^2^ qPCR in GSE-treated mouse 3T3-L1 differentiated adipocytes (Figure 3A).

To further substantiate the changes in the expression of adipogenesis-related genes by GSE, we next assessed the expression of effector protein molecules associated with adipogenesis in mouse 3T3-L1 adipocytes (Figure 3B). Our results indicated a differential modulation of adipogenesis-related proteins during different stages of adipocyte differentiation by GSE. While chronic GSE treatment in mouse 3T3-L1 adipocytes for 0–3 days increased the protein expression of C/EBP-α, PPAR-γ and FAS on day 3 of the adipocyte differentiation process, chronic GSE treatment for 8 days decreased the protein expression of C/EBP-α, C/EBP-β, SREBP-1/c, PPAR-γ and FAS in the matured adipocytes on day 8 (Figure 3B). Taken together, from these results, it was evident that though there was no change in the lipid content of adipocytes after chronic GSE treatment, GSE did modulate both mRNA and protein expression of the genes which are implicated in adipogenesis. Since an increase in UCP-1 mRNA levels also indicates towards the ‘browning’ of adipocytes [41–44] by GSE, we next confirmed this phenomenon by detecting whether mitochondriogenesis [an increase in mitochondria number which is an indicator of browning of adipocytes [41–44], was also increased in these adipocytes following GSE exposure (Figure 3C). Using MitoTracker Red staining of mitochondria, it was observed that GSE treatment indeed results in a significant increase in mitochondriogenesis in adipocytes (Figure 3C). Since the role of ‘brown adipocytes’ is different from white adipocytes [45–48], taken together, above results suggested a functional modification of adipocytes by GSE exposure towards a more ‘brown’ phenotype.

### GSE inhibits the growth promoting potential of adipocyte secretions on CRC cells

**Figure 4:**
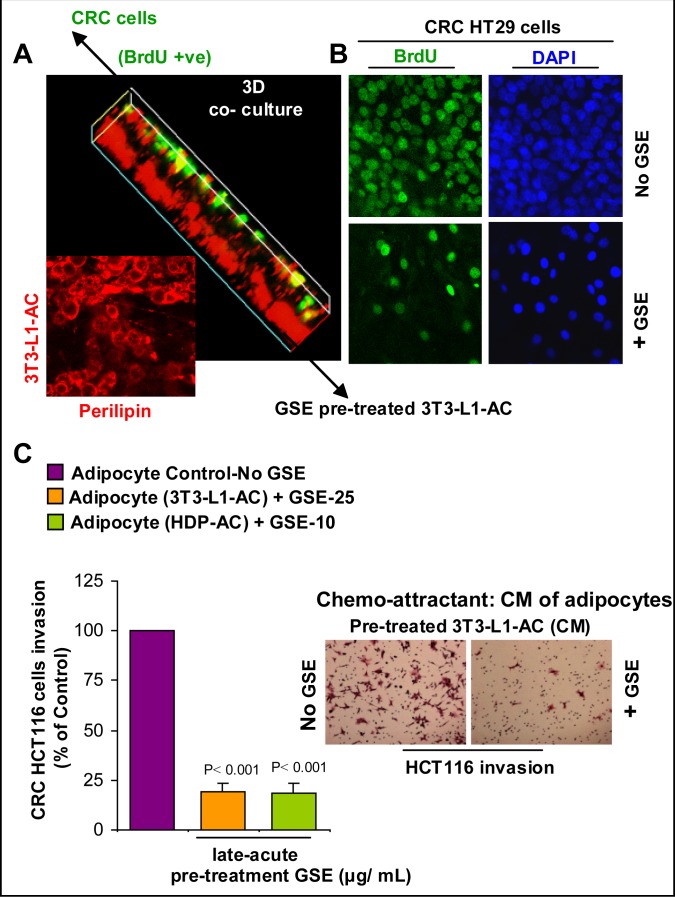
Effect of GSE on the growth promoting potential and chemotactic properties of adipocytes towards CRC cells **(A)** 3D model of adipocyte-CRC no-contact co-culture. Pre-adipocytes (3T3-L1 fibroblasts) were plated on the bottom of 8 well-BD-chamber slide and then allowed to differentiate in matrigel for 8 days. Mature adipocytes (3T3-L1-AC) were then treated with GSE (10 μg/ mL) for 2 days (late-acute treatment on day 8 for 48h). GSE was next washed off and the top of the matrigel was overlaid with a single layer of CRC HT29 cells in serum free media for 6 days. Cells were exposed to BrdU for 24 h, before study end; strong perilipin and BrdU –ve staining identified differentiated adipocytes. **(B)** Representative photomicrographs (X 200 magnification) indicate the decrease in the number of BrdU +ve cells-green, after 6 days of co-culture. DAPI-blue was used to stain nuclei. **(C)** GSE decreases the chemotactic properties of adipocytes towards HCT116 CRC cell invasion. Adipocyte-conditioned media (CM: collected for 48 h) after late-acute pre-treatment with GSE was used as the chemo-attractant in BD matrigel invasion chamber. After 48 h, matrigel invaded CRC cells were methanol fixed, H&E stained and counted. Adipocytes used were 3T3-L1-AC and human type II diabetic visceral adipocytes (HDP-AC). Representative photomicrographs of H&E stained (X 100 magnification) matrigel invaded HCT116 CRC cells are shown. Columns, mean values; error bars, SEM.

Since the predominant effect of GSE on adipocytes was their functional modification, we next focused our studies on assessing the biological consequences of this GSE effect on CRC cells. Specifically, to confirm that indeed GSE affects the growth promoting potential of adipocyte secretions on CRC cells, a 3D model of adipocyte-CRC no-contact co-culture was generated (Figure 4A). In this co-culture 3D system, mature adipocytes were pre-treated with GSE and then GSE was removed before co-culture of mature adipocytes with CRC cells, so that no direct effect of GSE on CRC cells could interfere in study outcomes. Briefly, pre-adipocytes (mouse 3T3-L1 fibroblasts) were plated in the bottom of 8 well-BD-chamber slide (Cat #354108, BD Falcon) and then allowed to differentiate in matrigel for 8 days. Mature mouse 3T3-L1 differentiated adipocytes were then treated with GSE (10 μg/mL) for 2 days (late-acute GSE treatment on day 8 for 48h). GSE was next washed off, and the top of the matrigel was overlaid with a single layer of CRC HT29 (Figure 4) or SW480 (data not shown) cells in serum free media for 6 days. Cells were exposed to BrdU for 24 h before study end (6 days) and the BrdU +ve CRC cells were counted (Figure 4B). Our results indicated a significant decrease in the proliferation of CRC cells (BrdU +ve cells) after 6 days of their no-contact co-culture with GSE-treated adipocytes compared to control adipocytes (Figure 4B). A strong perilipin (used for lipid staining) and BrdU –ve staining was used to identify the differentiated adipocytes at the bottom of the well in both untreated and GSE-treated adipocytes (Figure 4A). Importantly, these results show that the GSE effects were primarily due to functional modification in growth promoting signals of adipocytes on CRC cells and not due to changes in lipid content of matured adipocytes as evident from perilipin staining of lipids in the experiment and also based on our above results showing no changes in percent ORO staining intensity in adipocytes after GSE treatments (Figure 1).

### GSE decreases the chemotactic properties of adipocytes towards CRC cell invasion

To determine whether GSE also has the potential to decrease the chemotactic properties of adipocyte signaling towards CRC cells, we focused on a CRC cell line with known invasion and migratory characteristics (Figure 4C). Since HT29 and SW480 cells are non-invasive under *in vitro* conditions, HCT116 CRC cells with established invasive potential under *in vitro* conditions [49] were used. Briefly, adipocyte-conditioned media (collected at 48 h) after chronic or late-acute pre-treatment with GSE was used as the chemo-attractant (supplemented with 0.5% FBS) in the lower chamber of BD matrigel invasion chamber for HCT116 CRC cells (4 × 10^5^ cells in DMEM media containing 0.5% FBS) in the upper chamber. After 48 h, matrigel invaded CRC cells (Figure 4C) were methanol fixed, H&E stained and counted as described earlier [50]. Adipocytes used for collection of conditioned media were mature mouse 3T3-L1 differentiated adipocytes and human type II diabetic visceral adipocytes. GSE was used to pre-treat these adipocytes by adding the selected optimum doses during differentiation events [chronic treatment] or after adipocyte maturation [late-acute]. These treated adipocytes were then washed to clear off GSE, and then incubated with serum and GSE free media for 48 h so as to collect adipocyte-conditioned media which was later used as a chemo-attractant for CRC cells in BD matrigel invasion assay. Important to note here is that adipocytes were pre-treated with GSE and then GSE was removed before using these mature adipocytes as chemo-attractant for invasion of CRC cells, so that no direct effect of GSE on CRC cells could interfere in study outcomes. Cell counts of CRC cells that had invaded matrigel indicated that the invasion of HCT116 CRC cells was significantly reduced when conditioned media from both chronic (data not shown) and late-acute (Figure 4C) GSE-treated adipocytes was used. These results show that GSE effects were primarily due to functional modification of migratory /invasion promoting signals of adipocytes on CRC cells and not due to interference in differentiation of pre-adipocytes to mature adipocytes or the lipid content of mature adipocytes based on our above results showing no changes in percent ORO staining intensity in adipocytes after GSE treatments (Figure 1).

### GSE inhibits adipocyte mediated pro-tumorigenic signals on CSC enriched colonospheres

**Figure 5:**
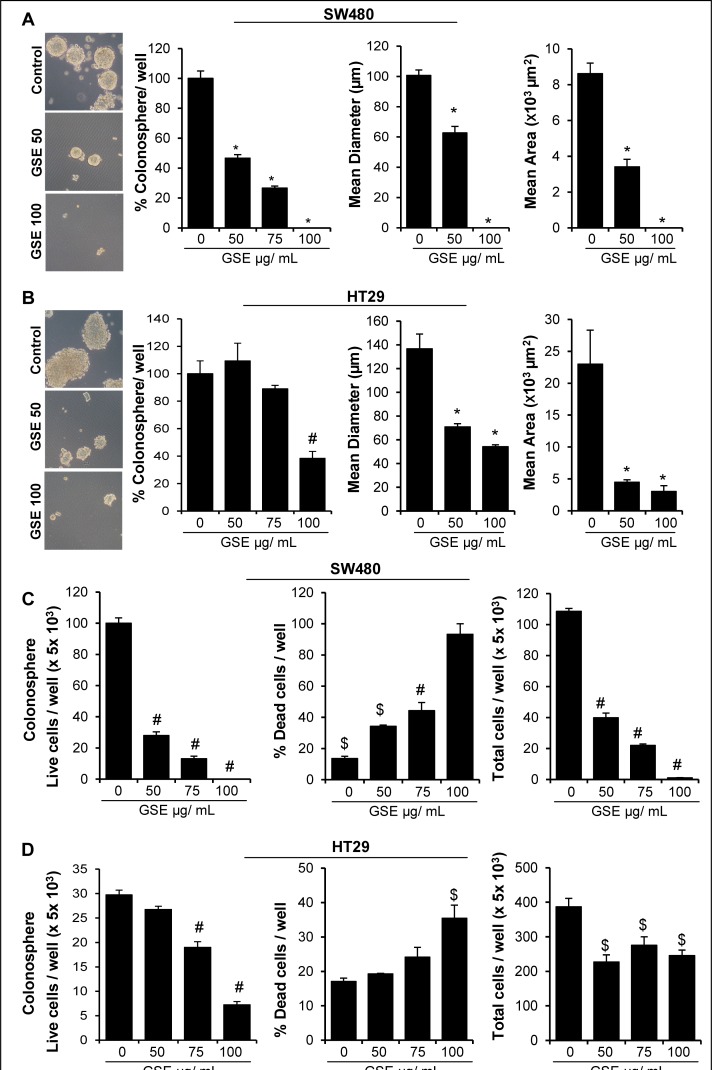
Effect of GSE on the formation of CSC enriched colonosphere in SW480 and HT29 CRC cells **(A-B)** Effect of different doses of GSE on number and size (diameter and area) of CSC enriched SW480 and HT29 colonospheres in sphere cluster formation assays after 10 days. A& B-*left panels*) Representative phase contrast photomicrographs (X 100 magnification, crop factor: 10) of CSC enriched colonospheres depicting a decrease in their size by GSE treatment. Area of colonospheres was measured using Zeiss Axioscope 2 microscope software (Carl Zeiss, Inc., Jena, Germany). $ *P*<0.05, # *P*<0.02; * *P*<0.001. **(C-D)** Effect of GSE on the viability of CRC cells in colonospheres. Colonospheres were dispersed as single cells by Accutase treatment, and Trypan blue dye exclusion assay was used to assess cell viability. Columns, mean values; error bars, SEM.

Recent studies have identified CD44^+^EpCAM^high^ cells as the tumor cell populations harboring CSC properties (self-renewal and aberrant differentiation) in CRC, which drive tumorigenic events [16, 51, 52]. Accordingly, we performed the studies to assess both the direct effect of GSE as well as that of pro-tumorigenic signals arising from the GSE-treated adipocytes secretions, on the self-renewal capacity of CSC population in CRC cell lines. For the first set of studies, we isolated CSC enriched CD44^+^EpCAM^high^ cell populations from human CRC cell lines SW480 and HT29 [53] and then subjected them to sphere cluster formation assays (Figure 5) [53] in the absence or direct presence of GSE (50–100μg/mL), and % of floating colonospheres (≥ 50 cells per sphere) generated after 10 days were determined (Figure 5A-B). Whereas a single GSE treatment significantly decreased the number of SW480 colonospheres in a dose-dependent manner, the inhibitory effect was significant only at 100μg/mL GSE dose in HT29 colonospheres (Figure 5A). Furthermore, we calculated the diameter and area of individual colonospheres and the results showed a significant decrease in these parameters after GSE exposure (at all doses in both cell lines) which indicated that GSE treatment had an inhibitory effect on the progenitor/ bulk daughter CRC cells in the colonospheres (Figure 5A-B). The growth inhibitory effects of GSE in these colonospheres were further confirmed by measuring cellular viability of dissociated spheres using Trypan blue dye exclusion assay (Figure 5C-D). The results showed that GSE caused a significant decrease in live as well as total cell numbers with a strong increase in the number of dead cells in both SW480 and HT29 colonospheres (Figure 5C-D).

**Figure 6:**
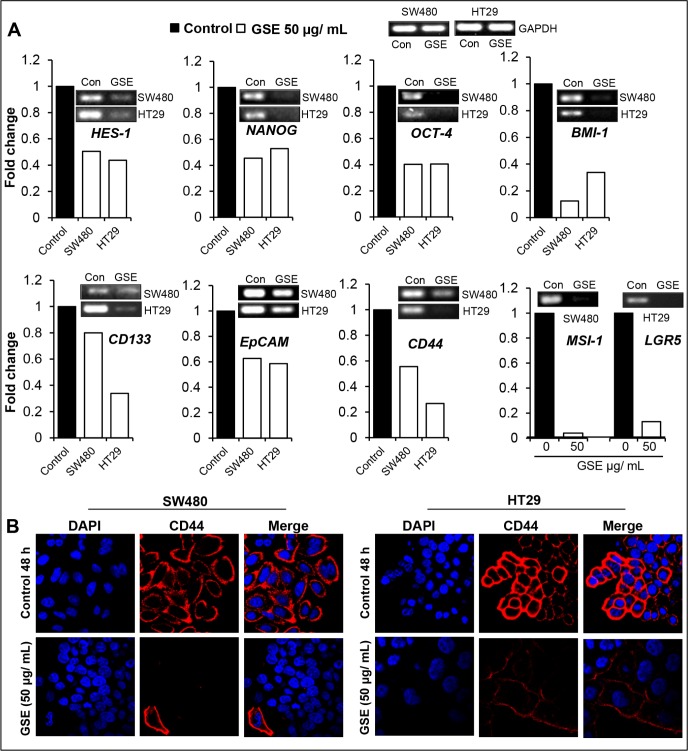
Effect of GSE on mRNA levels of CSC associated-signaling molecules, transcription factors, markers, and CD44 protein levels in CRC cells **(A)** Effect of GSE on *HES-1, NANOG, OCT-4, BMI-1, CD133*, *EpCAM, CD44, MSI-1 and LGR5* mRNA levels in HT 29 and SW480 CRC cells as determined by semi-quantitative RT-PCR analysis. Columns indicate fold changes relative to untreated controls and were normalized to *GAPDH* mRNA levels. **(B)** Effect of GSE on the protein expression of CD44 in SW480 and HT29 CRC cells as observed by immunofluorescence staining. Representative photomicrographs of immunofluorescence staining (X 600 x 2 magnification) of CRC cells with CD44-red and DAPI-blue as nuclear stain are shown. GSE dose (50 μg/mL, for 48h).

To examine whether GSE efficacy against colon CSCs involves an alteration in the gene levels of CSC associated-signaling molecules, -transcription factors, and -markers, we next performed semi-quantitative RT-PCR analysis on selected colon CSC associated genes after 48h of GSE exposure in monolayer cell culture of CRC cells (Figure 6A). Indeed, GSE treatment down regulated the mRNA levels of: a) CSC regulatory molecule *HES-1* that is the transcriptional target of NOTCH 1 signaling, and b) CSC-associated transcription factors and markers, namely *NANOG, OCT-4, BMI-1, CD133, EpCAM, CD44, MSI-1 and LGR5* in both SW480 and HT29 cell lines (Figure 6A). Based on the data showing that GSE modulated the mRNA expression of CD44, the CSC associated-marker, next we also assessed the protein expression of CD44 in the CRC cell lines (Figure 6B). Immunofluorescence staining for CD44 indicated a dramatic decrease in the expression of membrane CD44 in both SW480 and HT29 CRC cell lines after GSE treatment (Figure 6B).

**Figure 7:**
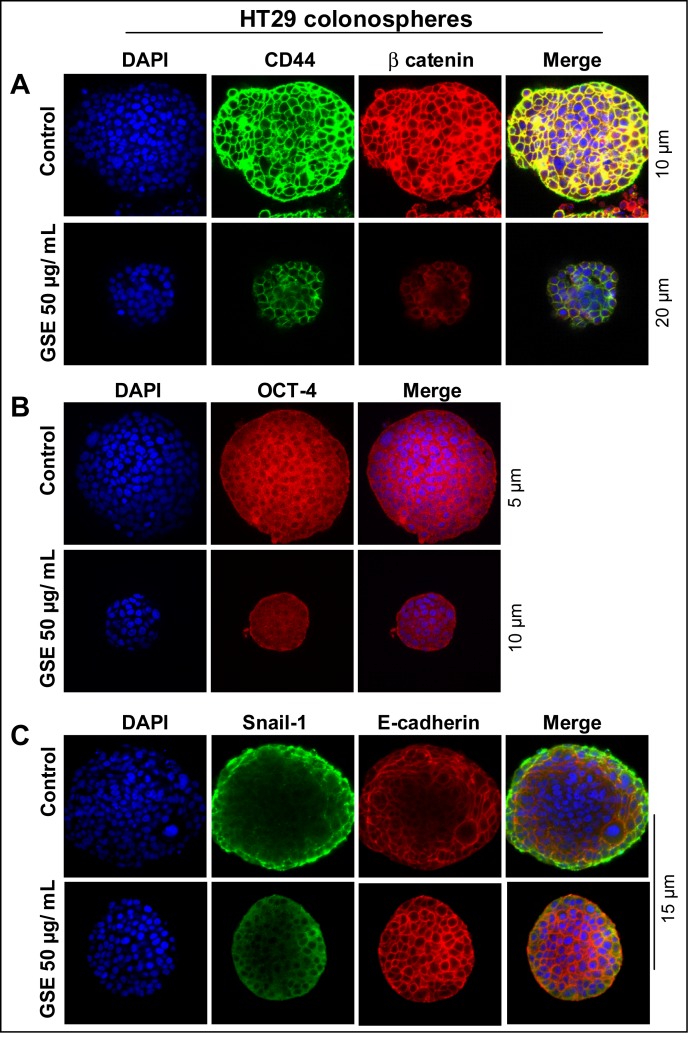
Effect of GSE on the protein expression of CD44, β-catenin, Oct-4, SNAIL-1 and E-cadherin in CSC enriched CRC colonospheres Effect of GSE on the protein expression of, **(A)** CD44 and β-catenin; **(B)** OCT-4; and **(C)** SNAIL-1 and E-cadherin levels in CSC enriched HT29 colonospheres. Stained samples were mounted with DAPI containing anti-fade reagent (Invitrogen) for visualization under confocal microscopy. Z stack analysis was performed and representative scans (X 600 magnification) with individual scan depth of specific colonospheres representing highest fluorescence intensity/signal are shown.

Since CD44 is an essential CSC marker [16, 51, 52, 54], next, we also performed the Z stack analysis (Figure 7A) of HT29 colonospheres, to determine how protein expression of this marker and another essential colon CSC-associated regulatory molecule, β-catenin [55], was modulated by GSE. Similar to our data in monolayer culture (Figure 6), we found a drastically lower expression of both CD44 and β-catenin in GSE-treated colonospheres compared to controls (Figure 7A). Similarly, the expression of OCT-4 was also effectively reduced by GSE (Figure 7B). Since CSCs have been associated with increased migratory and invasive properties, we next also evaluated the colonospheres for the expression of a) SNAIL-1, a mesenchymal marker, the increased expression of which is associated with migratory characteristics of cells, and b) E-cadherin, the epithelial localization of which imparts epithelial differentiation and its increased expression causes the cells to lose their migratory potential [56–58]. The Z scans showing the staining patterns for these molecules indicated that while there was an increased expression of SNAIL-1 in the periphery of control colonospheres with less overall E-cadherin staining, the SNAIL-1 staining was drastically decreased while membrane E-cadherin expression was significantly increased in all the cells of GSE-treated colonospheres (Figure 7C).

**Figure 8:**
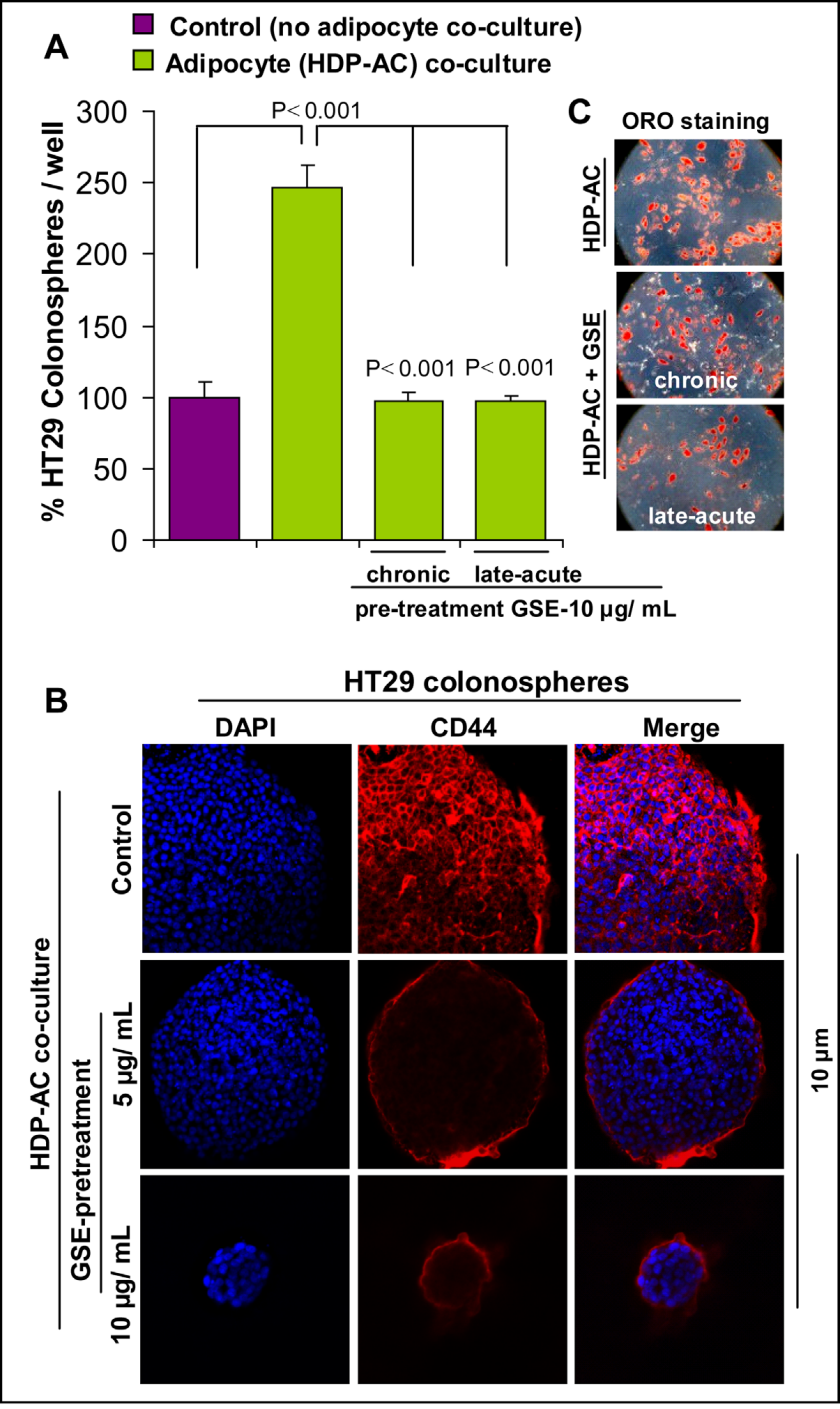
Effect of GSE on CSC enriched colonosphere formation, induced by no-contact co-culture with adipocytes Human type II diabetic visceral pre-adipocytes were grown on cover slips and allowed to differentiate and mature in to human type II diabetic visceral adipocytes (HDP-AC). GSE (10 μg/mL) was used to pre-treat these adipocytes by adding it during differentiation events [chronic treatment: 0-12 days] or after adipocyte maturation [late-acute: day 12 for 48 h]. **(A)** Matured adipocytes with or without GSE pre-treatment were then used in no-contact HT29 colonosphere assays. After 10 days, the number and size of colonosphere formed was measured. **(B)** Effect of GSE on the protein expression of CD44 in HDP-AC mediated CSC enriched HT29 colonospheres as observed by immunofluorescence staining. Representative z scan images of immunofluorescence staining (X 400 magnification using confocal microscopy) of colonospheres with CD44-red and DAPI-blue as nuclear stain are shown. Scan depth of specific colonospheres are also shown. **(C)** Representative photomicrographs (X 200 magnification) of Oil-Red-O (ORO) based identification of differentiated HDP-AC (red lipid droplets), in the absence and presence of GSE exposure are shown. Columns, mean values; error bars, SEM.

Next, in the second set of the studies, to mimic the physiological influence of tumorigenic signals arising from the adipocyte secretions (systemic as well as peritoneal), colonosphere formation assay was performed in the presence of mature adipocytes (no-contact co-culture) (Figure 8A). Briefly, human type II diabetic visceral pre-adipocytes were grown on cover slips and allowed to differentiate and mature in to human type II diabetic visceral adipocytes. GSE (10 μg/mL, optimum dose) was used to pre-treat these adipocytes by adding it during differentiation events [chronic treatment: 0–12 days] or after adipocyte maturation [late-acute: at day 12 for 48 h]. Matured adipocytes with or without GSE pre-treatment on cover slips were then washed to clear off GSE and placed at the bottom of low attachment six-well plates. These adipocytes on cover slips were then incubated for 48 h with serum-free stem cell specific media to be used in colonosphere assays. After 48 h, colon CSCs were seeded as single cells (3000 cells/well) in this media for colonosphere formation. It is important to emphasize here that it was a no contact co-culture model as adipocytes on cover slips were placed on the bottom of wells whereas the colonospheres in the sphere cluster formation assays are generated as floating spheroids in the media and are not in contact with the adipocytes but at the same time are exposed to adipocytes secretions. After 10 days, the number and size of colonosphere formed was measured (Figure 8A). Important to note here is that adipocytes were pre-treated with GSE and then GSE was removed before co-culture of mature adipocytes with colon CSC, so that no direct effect of GSE on colon CSC could interfere in study outcomes.

Our results indicated that compared to control colon CSC alone, presence of adipocytes caused a significant increase in HT29 colonospheres formation; however, chronic or acute GSE pre-treatment of adipocytes significantly reduced the booster signals of adipocytes resulting in decreased colonosphere numbers that were comparable to those without adipocytes (Figure 8A). The Z stack analysis of the colonospheres was next performed (Figure 8B), which revealed that protein expression of CD44 was markedly decreased in the CSC enriched colonospheres which were co-cultured in the presence of GSE-exposed adipocytes compared to untreated adipocytes. Also importantly, these results showed that GSE effects were primarily due to functional modification of growth promoting signals of adipocyte secretions on colon CSC and not due to interference in differentiation of pre-adipocytes to mature adipocytes or in the lipid content of mature adipocytes as indicated by ORO staining based identification (Figure 8C) of differentiated human type II diabetic visceral adipocytes (red lipid droplets).

## DISCUSSION

Recent studies have ascribed the major reason for the failure of most of the treatment strategies against CRC to the presence of CSC population in the colon tumor mass, which is essentially resistant to current therapeutic strategies, compared to daughter cells in the bulk tumor cell population [12–18]. As stem cells or their progenitors are the targets of transformation into CSCs which are responsible for tumorigenesis [13–15, 17, 18], etiological factors that interfere with the pro-tumorigenic signals affecting the CSC population needs to be identified. Since obesity is associated with increased CRC incidence and mortality [8], it is imperative that obesity-driven triggers that cause enhanced colon tumorigenesis, due to the expansion of CSC pool, be critically defined. Recently, there is a paradigm shift that adipose tissue surrounding epithelial tumors are not ‘idle-bystanders’, but rather they actively participate in signaling that alters microenvironment in favor of tumor growth and progression [9–11]. However, not much is known about the impact of adipose tissue-modified microenvironment on both CRC and colon CSC; the latter being now recognized as the real driving force behind CRC initiation, promotion and progression [12, 14, 16, 59]. Such an effort is now not only important but rather essential since obese conditions play an important role in increased CRC growth [3, 7, 8]. In light of these considerations, there is an urgent need to understand the crucial role of adipocytes secretions (both in systemic circulation and peritoneal fluids) on colon CSC that drives CRC under obese conditions. Though the role of adipocytes, the major players in obesity, on colon carcinogenesis is just beginning to unfold, nevertheless in the growing obesity epidemic, it is essential that we investigate the benefits of those agents which exert both anti-obesogenic and anti-CRC effects resulting in CRC prevention and control under obese conditions.

In the present study, employing a panel of mouse and human (diseased and non-diseased) adipocytes, we found that GSE pre-treatment of adipocytes (in co-culture under no contact model using a 3D matrigel culture system) decreases their growth promoting effects on CRC cells. In addition, adipocyte-conditioned media, collected after chronic and acute pre-treatment with GSE, significantly decreased the chemotactic properties of adipocytes towards CRC cell invasion. Furthermore, not only did GSE alone exert inhibitory effect on the self-renewal capacity of the colon CSC population as evidenced by a decrease in both the number and size of colonospheres and altered mRNA/protein levels of various CSC-associated markers and regulatory molecules, the growth promoting signals of adipocytes on colon CSC population were also dramatically reduced by GSE. Notably, these effects of GSE on adipocytes were not associated with any changes in the lipid content of adipocytes after GSE exposure. However, GSE was found to increase mitochondriogenesis and *UCP-1* mRNA levels together with differential modulation of adipogenesis-related genes/proteins during different stages of adipocyte differentiation. Since increased mitochondriogenesis and UCP-1 expression are associated with brown adipocytes [41–47], we presumed that somehow GSE treatment was causing the ‘browning’ of adipocytes and thereby interfering with the pro-tumorigenic signals of adipocytes on colon CSCs/CRC cells.

The above observations and interpretations have strong implications, given the fact that white and brown adipocytes have functionally different roles. Briefly, adipose tissue in mammals exists in two distinct forms, namely WAT and BAT [45–48, 60]. WAT is composed of white adipocytes which store excess energy in the form of triglycerides; however, BAT is primarily made up of brown adipocytes which uniquely express UCP-1; activation of UCP-1 uncouples the proton gradient in the mitochondria from ATP generation which results in dissipation of energy as heat [45–48, 60]. Furthermore, WAT has relatively few mitochondria and secretes a variety of hormones and adipokines, but BAT burns lipids to generate heat and expend energy and is involved in plasma triglyceride clearance and glucose homeostasis. This underlies the importance of BAT in obesity and related metabolic disorders [45, 46]. Unlike rodents, in humans, the prevalence of BAT decreases in adulthood, thus its functionality in human adults is limited and is further diminished in obese individuals [45–47]. However, in recent years, the existence of adipocytes with an intermediate phenotype between brown and white, known as ‘brite’ adipocytes, indicates a possibility of ‘browning’ of WAT to a ‘brown phenotype’ with beneficial effects [45–48, 60]. The ‘brite’ adipocytes are reminiscent of a lean adipose tissue, which is neither associated with increased secretion of pro-inflammatory adipokines and cytokines from the adipocytes and inflammatory cells, respectively, nor involved in tumor development and promotion [4, 11, 45–47]. Thus, strategies to increase both activity and recruitment of these ‘brite’ adipocytes are currently being investigated and recognized as a new therapeutic/preventive target to combat obesity-associated disorders [41–47].

Taken together above discussion and our findings that GSE efficacy against CRC under obese conditions may possibly be mediated *via* its ability to induce ‘brown remodeling’ of white adipocytes, GSE stands out as a strong candidate agent for both pre-clinical and clinical studies in future for its potential use against CRC growth and progression under obese conditions. Furthermore, extensive investigations into the molecular mechanisms of GSE potential to induce the browning phenomenon in adipocytes are also required and will be the focus of our future studies. Successful outcomes from such studies would have significant clinical impact on rationalized use of GSE to control obesity-associated human CRC in particular and other malignancies in general.

## MATERIALS AND METHODS

### Reagents

Standardized preparation of GSE [22–24] was a gift from Kikkoman Corp. (Nado City, Japan) and was dissolved in DMSO for cell culture use. Antibodies used were: CD44 total (Cat # sc-65410), C/EBP-α (Cat # 2295), C/EBP-β (Cat # 3082), FAS (Cat # 3180) and SREBP-1c (Cat # 366), and PPAR-γ (Cat # sc-7273) from Santa Cruz Biotechnology; BrdU-FITC (Cat # ab74545) and OCT-4 (Cat # ab18976) from Abcam; CD44-FITC (Cat # 555478, BD Pharmingen); EpCAM-PE (Cat # 347211BD Biosciences); β-catenin (Cat # 9582), Snail-1 (Cat # 3895), E-cadherin (Cat # 3195), and Perilipin (Cat # 9349) from Cell Signaling Technology. Anti-β-actin (Cat # A2228) antibody was from Sigma. Secondary antibodies, anti-rabbit IgG-HRP-linked (Cat # 7074) and anti-mouse IgG-HRP-linked (Cat # 7076) were from Cell Signaling Technology. Texas Red (Cat # T2767) or Alexa Flour 488 (Cat # A11008) or Alexa Fluor 594 (Cat # A110032) conjugated secondary antibodies and BrdU labeling reagents (Cat # 00-0103) were from Invitrogen. BODIPY 493/503 (Cat # D-3922) and MitoTracker Red (Cat# M-7152) were from Molecular Probes. Growth factor (GF) reduced matrigel was from BD Bioscences (Cat # 356231). Invasive potential of CRC cells was determined using matrigel coated trans-well chambers (8 micron pore size) from BD Biosciences. Trizol^R^ method was used to isolate total RNA from CRC cells/ adipocytes, first-strand cDNA prepared using RT-PCR first strand kit (Cat # 330401,Qiagen), and semi-quantitative RT-PCR analysis was done using specific primers from Sigma as described earlier [53]. Primer sequences are detailed in Supplementary Table 1. For RT^2^qPCR analysis of adipogenesis related genes, RT^2^ Profiler^TM^ PCR Array (Qiagen) for mouse (Cat # PAMM-049ZA) and human (Cat # PAHS-049ZA) was employed, while for RT^2^qPCR analysis of mouse *Ucp-1* gene, Qiagen kit (Cat # PPM05164B) was used. All RT^2^ qPCR analysis were done using ABI 7500 cycler as described earlier [53].

### Cell lines

SW480, HT29 and HCT116 cells were from ATCC (Manassas, VA), and maintained under 37°C /5% CO_2_ conditions in DMEM (HT29, and HCT116,) and RMPI (SW480) media supplemented with 10% FBS and 1% penicillin/streptomycin (Cat #15140, Gibco). Mouse 3T3-L1 differentiated adipocytes (3T3-L1-AC) were generated from mouse 3T3-L1 fibroblasts (ATCC). Human differentiated adipocytes were generated from well-defined human adipose tissue derived mesenchymal stem cells (Cat #PCS-500-011, ATCC) and human visceral pre-adipocytes associated with type II diabetes (Cat # PT-5008, Lonza Group Ltd.); these differentiated adipocytes are referred in the figures as MSC-AC and HDP-AC, respectively.

### Adipocyte Culture and Differentiation

Well-defined differentiation protocols were followed, as shown in supplementary figure 1. Mouse 3T3-L1 fibroblasts were cultured and maintained in a growth media consisting of high glucose DMEM (Cat #11995, Gibco) and 1% penicillin-streptomycin, supplemented with 10% bovine calf serum (Cat #30-2030, ATCC). The 3T3-L1 adipocyte differentiation was induced at 2 days post-confluency (designated as day 0) by exposing confluent fibroblasts (pre-adipocytes) to an adipogenic induction media, consisting of growth media [high glucose DMEM, 1% penicillin-streptomycin and 10% FBS (Cat #10437, Gibco)], supplemented with 1 μM dexamethasone (Cat #API-04, G-Biosciences), 0.5 mM isobutylmethylxanthine (IBMX, Cat #I5879, Sigma), and 1 μg/mL bovine insulin (Cat #128-100, Cell Applications, Inc.) for 3 days. On day 3, the adipogenic induction media was replaced with media consisting of the growth media supplemented with 1 μg/mL bovine insulin [without IBMX and dexamethasone] and after another 3 days, on day 6, the adipocytes were maintained in growth media only (without bovine insulin, IBMX and dexamethasone).

Human visceral pre-adipocytes associated with type II diabetes were cultured and differentiated in to adipocytes according to manufacturer's protocol. Briefly, after reaching confluency, human visceral pre-adipocytes were incubated with adipogenic differentiation media (Cat #PT-8002, Lonza) for 12 days for differentiation into mature adipocytes. Human adipose tissue-derived mesenchymal stem cells were cultured and induced to differentiate into adipocytes according to differentiation kit provided by ATCC (Cat #PCS-500-050). Briefly, adipose-derived mesenchymal stem cells were incubated with adipogenic differentiation initiation media [15 mL of adipocyte basal medium and 1 mL of adipocyte differentiation (AD) supplement] for 4 days (media replenished after 2 days) and then differentiated cells were maintained in adipocyte differentiation maintenance medium [85 mL of adipocyte basal medium and 5 mL of adipocyte differentiation maintenance (ADM) supplement] for another 10 days (media changed after every 3 days) for differentiation into mature adipocytes.

### Protocols for GSE treatment

Since adipocyte differentiation time varied between different cell lines, the specific treatment schedule (Supplementary Figure 1) for chronic and late-acute GSE treatments are detailed as: **1a)** Chronic treatments in 3T3-L1-AC: GSE added at day 0 (induction of adipogenesis in post-confluent pre-adipocytes) till day 8 (day of adipocytes maturation); **1b)** Late-acute treatments in 3T3 L1-AC: GSE added at day 8 (day of adipocytes maturation) for 48 h; **2a)** Chronic treatments in human type II diabetic visceral adipocytes: GSE added at day 0 (induction of adipogenesis in post-confluent pre-adipocytes) till day 12 (day of adipocytes maturation); **2b)** Late-acute treatments in human type II diabetic visceral adipocytes: GSE added at day 12 (day of adipocytes maturation) for 48 h; **3a)** Chronic treatments in human adipocytes from adipose tissue derived mesenchymal stem cells: GSE added at day 0 (induction of adipogenesis in post-confluent pre-adipocytes) till day 14 (day of adipocytes maturation); and **3b)** Late-acute treatments in human adipocytes from adipose tissue derived mesenchymal stem cells: GSE added at day 14 (day of adipocytes maturation) for 48 h. It should be noted that during chronic GSE treatments, wherever media changes were required during the adipogenesis process, following removal of spent media, the fresh media that was added to the cells was supplemented with the required concentration of GSE. Adipocyte-conditioned media was obtained by subjecting specific adipocytes to serum free media for 48 h, and then using the cell clarified, generated sterile media, in subsequent *in vitro* experiments in place of regular media. Total cellular lysates, determination of protein concentrations and western blotting followed by ECL detection were done as described previously [23, 24].

### Staining of lipids and mitochondria

ORO staining was performed to identify intracellular lipid droplets in matured and differentiated adipocytes. Briefly, ORO stock solution was prepared by dissolving 300 mg ORO powder in 100 mL of 100% isopropanol. As working solution, 3 parts of ORO stock was mixed with 2 parts of water and allowed to sit at room temperature (RT) for 10 min and then filtered. The working solution was prepared fresh before use. At the end of adipocyte differentiation events, the adipocytes were washed with PBS, formalin fixed for 1 h, washed with 60% isopropanol and then dried in air at RT before exposing them to ORO solution for 15 min. ORO stained cells were rinsed with water four times to wash of excess stain and observed under microscope to identify red stained lipid droplets. For quantification purposes, ORO stained cells were eluted with 100% isopropanol for 10 min and absorbance of the elutes was measured at O.D. 500 nm. Lipids were also stained in formalin fixed cells by using either directly green fluorescent BODIPY 493/503 (1μg/mL) dye or anti-perilipin primary antibody (1:200) followed by texas red conjugated goat anti-rabbit-secondary antibody. The slides were mounted with Prolong Gold Antifade reagent with DAPI and observed under confocal microscope. For mitochondria detection, live cells were exposed to MitoTracker Red dye (100 nM) in serum free media for 30 min at 37^°^C, washed and formalin fixed; following mounting with DAPI, thread like bright red stained organelles were identified as mitochondria under confocal microscope.

### Sphere cluster formation assays

CRC cell populations were subjected to sphere cluster formation assays as described earlier [53]. Briefly, single sorted FACS separated CD44^+^EpCAM^high^ cells [employing CD44-FITC and EpCAM-PE antibodies] were cultured (3000 cells/ well) in stem cell specific serum free media (2mL) in an ultra-low attachment six well plates (Cat # 3471, Costar) for 10–12 days. The defined stem cell specific serum free media was DMEM/F-12 (Cat #21041-025) supplemented with 1% penicillin-streptomycin, B27 (Cat # 17504-044) and N2 (Cat #17502-048) supplements (all from Gibco), and growth factors [recombinant human epidermal growth factor (Cat # 01-107) and fibroblast growth factor (Cat#PHG0261), both from Invitrogen]. GSE was added after 6 h of seeding. Colonospheres with ≥ 50 cells were scored as true colonospheres. To measure cellular viability in colonospheres, spheres were dissociated with Accutase for 15 min at 37^o^C and subjected to Trypan blue dye exclusion assay

### Microscopic and statistical analysis

In invasion assay, matrigel invaded CRC cells were manually counted at 400X [50]. For Immunofluorescence staining of colonospheres, the colonospheres were harvested and immobilized for staining convenience as described by us recently [53]. Fluorescent images of cells / colonospheres were captured using a Nikon D-Eclipse C1 confocal microscope (Nikon) and analyzed using EZ-C1 Free viewer software. Z stacking was performed for colonospheres, as detailed recently [53]; for comparisons, averaged interval scans that best represent the highest fluorescence intensity/signal were used. In treatment assays, difference between treatment and control groups was determined by one-way ANOVA or un-paired two-tailed Student's *t-*test using Sigma stat 2.03 software. Two-sided P values of (0.05 were considered significant. For western blots, β-actin was used as loading control after stripping and re-probing the blots, and densitometric values were calculated by Image J (NIH) software using correction factor for β-actin blots.

## SUPPLEMENTARY FIGURE AND TABLE



## ABBREVIATIONS

GSE: grape seed extract
CRC: colorectal cancer
CSC: cancer stem cells
3T3-L1-AC: mouse 3T3-L1 differentiated adipocytes
MSC-AC: human adipocytes from adipose tissue-derived mesenchymal stem cells
HDP-AC: human type II diabetic visceral adipocytes
3D: 3 dimensional
ORO: Oil-Red-O

## References

[1] National Cancer Institute: PDQ® Genetics of Colorectal Cancer http://cancer.gov/cancertopics/pdq/genetics/colorectal/HealthProfessional.

[2] Center MM, Jemal A, Ward E (2009). International trends in colorectal cancer incidence rates. Cancer epidemiology, biomarkers & prevention: a publication of the American Association for Cancer Research, cosponsored by the American Society of Preventive Oncology.

[3] Donohoe CL, Pidgeon GP, Lysaght J, Reynolds JV (2010). Obesity and gastrointestinal cancer. The British journal of surgery.

[4] Gregor MF, Hotamisligil GS (2011). Inflammatory mechanisms in obesity. Annual review of immunology.

[5] Hotamisligil GS (2006). Inflammation and metabolic disorders. Nature.

[6] Pais R, Silaghi H, Silaghi AC, Rusu ML, Dumitrascu DL (2009). Metabolic syndrome and risk of subsequent colorectal cancer. World journal of gastroenterology: WJG.

[7] Reynolds JV, Donohoe CL, Doyle SL (2011). Diet, obesity and cancer. Irish journal of medical science.

[8] Gallagher EJ, LeRoith D (2013). Epidemiology and molecular mechanisms tying obesity, diabetes, and the metabolic syndrome with cancer. Diabetes care.

[9] Matafome P, Santos-Silva D, Sena CM, Seica R (2013). Common mechanisms of dysfunctional adipose tissue and obesity-related cancers. Diabetes/metabolism research and reviews.

[10] Nieman KM, Romero IL, Van Houten B, Lengyel E (2013). Adipose tissue and adipocytes support tumorigenesis and metastasis. Biochimica et biophysica acta.

[11] Yehuda-Shnaidman E, Schwartz B (2012). Mechanisms linking obesity, inflammation and altered metabolism to colon carcinogenesis. Obesity reviews: an official journal of the International Association for the Study of Obesity.

[12] Boman BM, Huang E (2008). Human colon cancer stem cells: a new paradigm in gastrointestinal oncology. J Clin Oncol.

[13] Huang EH, Wicha MS (2008). Colon cancer stem cells: implications for prevention and therapy. Trends Mol Med.

[14] Lobo NA, Shimono Y, Qian D, Clarke MF (2007). The biology of cancer stem cells. Annu Rev Cell Dev Biol.

[15] McDonald SA, Graham TA, Schier S, Wright NA, Alison MR (2009). Stem cells and solid cancers. Virchows Arch.

[16] Ricci-Vitiani L, Fabrizi E, Palio E, De Maria R (2009). Colon cancer stem cells. J Mol Med.

[17] Sanchez-Garcia I, Vicente-Duenas C, Cobaleda C (2007). The theoretical basis of cancer-stem-cell-based therapeutics of cancer: can it be put into practice?. Bioessays.

[18] Ward RJ, Dirks PB (2007). Cancer stem cells: at the headwaters of tumor development. Annu Rev Pathol.

[19] Derry MM, Raina K, Agarwal C, Agarwal R (2013). Identifying molecular targets of lifestyle modifications in colon cancer prevention. Frontiers in oncology.

[20] Kaur M, Agarwal C, Agarwal R (2009). Anticancer and cancer chemopreventive potential of grape seed extract and other grape-based products. The Journal of nutrition.

[21] Rajamanickam S, Agarwal R (2008). Natural products and colon cancer: current status and future prospects. Drug Dev Res.

[22] Yamakoshi J, Saito M, Kataoka S, Kikuchi M (2002). Safety evaluation of proanthocyanidin-rich extract from grape seeds. Food and chemical toxicology: an international journal published for the British Industrial Biological Research Association.

[23] Kaur M, Mandair R, Agarwal R, Agarwal C (2008). Grape seed extract induces cell cycle arrest and apoptosis in human colon carcinoma cells. Nutrition and cancer.

[24] Derry M, Raina K, Agarwal R, Agarwal C (2013). Differential effects of grape seed extract against human colorectal cancer cell lines: The intricate role of death receptors and mitochondria. Cancer letters.

[25] Derry MM, Raina K, Agarwal R, Agarwal C (2014). Characterization of azoxymethane-induced colon tumor metastasis to lung in a mouse model relevant to human sporadic colorectal cancer and evaluation of grape seed extract efficacy. Experimental and toxicologic pathology: official journal of the Gesellschaft fur Toxikologische Pathologie.

[26] Derry MM, Raina K, Balaiya V, Jain AK, Shrotriya S, Huber KM, Serkova NJ, Agarwal R, Agarwal C (2013). Grape seed extract efficacy against azoxymethane-induced colon tumorigenesis in A/J mice: interlinking miRNA with cytokine signaling and inflammation. Cancer Prev Res (Phila).

[27] Derry MM, Somasagara RR, Raina K, Kumar S, Gomez J, Patel M, Agarwal R, Agarwal C (2014). Target identification of grape seed extract in colorectal cancer using drug affinity responsive target stability (DARTS) technique: role of endoplasmic reticulum stress response proteins. Current cancer drug targets.

[28] Huang S, Yang N, Liu Y, Gao J, Huang T, Hu L, Zhao J, Li Y, Li C, Zhang X (2012). Grape seed proanthocyanidins inhibit colon cancer-induced angiogenesis through suppressing the expression of VEGF and Ang1. International journal of molecular medicine.

[29] Kaur M, Singh RP, Gu M, Agarwal R, Agarwal C (2006). Grape seed extract inhibits in vitro and in vivo growth of human colorectal carcinoma cells. Clinical cancer research: an official journal of the American Association for Cancer Research.

[30] Kaur M, Tyagi A, Singh RP, Sclafani RA, Agarwal R, Agarwal C (2011). Grape seed extract upregulates p21 (Cip1) through redox-mediated activation of ERK1/2 and posttranscriptional regulation leading to cell cycle arrest in colon carcinoma HT29 cells. Molecular carcinogenesis.

[31] Velmurugan B, Singh RP, Agarwal R, Agarwal C (2010). Dietary-feeding of grape seed extract prevents azoxymethane-induced colonic aberrant crypt foci formation in fischer 344 rats. Molecular carcinogenesis.

[32] Velmurugan B, Singh RP, Kaul N, Agarwal R, Agarwal C (2010). Dietary feeding of grape seed extract prevents intestinal tumorigenesis in APCmin/+ mice. Neoplasia.

[33] Charradi K, Sebai H, Elkahoui S, Ben Hassine F, Limam F, Aouani E (2011). Grape seed extract alleviates high-fat diet-induced obesity and heart dysfunction by preventing cardiac siderosis. Cardiovascular toxicology.

[34] Decorde K, Teissedre PL, Sutra T, Ventura E, Cristol JP, Rouanet JM (2009). Chardonnay grape seed procyanidin extract supplementation prevents high-fat diet-induced obesity in hamsters by improving adipokine imbalance and oxidative stress markers. Molecular nutrition & food research.

[35] Ohyama K, Furuta C, Nogusa Y, Nomura K, Miwa T, Suzuki K (2011). Catechin-rich grape seed extract supplementation attenuates diet-induced obesity in C57BL/6J mice. Annals of nutrition & metabolism.

[36] Park SH, Park TS, Cha YS (2008). Grape seed extract (Vitis vinifera) partially reverses high fat diet-induced obesity in C57BL/6J mice. Nutrition research and practice.

[37] Ardevol A, Motilva MJ, Serra A, Blay M, Pinent M (2013). Procyanidins target mesenteric adipose tissue in Wistar lean rats and subcutaneous adipose tissue in Zucker obese rat. Food chemistry.

[38] Pinent M, Blade C, Salvado MJ, Blay M, Pujadas G, Fernandez-Larrea J, Arola L, Ardevol A (2006). Procyanidin effects on adipocyte-related pathologies. Critical reviews in food science and nutrition.

[39] Quesada H, del Bas JM, Pajuelo D, Diaz S, Fernandez-Larrea J, Pinent M, Arola L, Salvado MJ, Blade C (2009). Grape seed proanthocyanidins correct dyslipidemia associated with a high-fat diet in rats and repress genes controlling lipogenesis and VLDL assembling in liver. Int J Obes (Lond).

[40] Charradi K, Elkahoui S, Karkouch I, Limam F, Hassine FB, Aouani E (2012). Grape seed and skin extract prevents high-fat diet-induced brain lipotoxicity in rat. Neurochemical research.

[41] Festuccia WT, Blanchard PG, Deshaies Y (2011). Control of Brown Adipose Tissue Glucose and Lipid Metabolism by PPARgamma. Frontiers in endocrinology.

[42] Petrovic N, Walden TB, Shabalina IG, Timmons JA, Cannon B, Nedergaard J (2010). Chronic peroxisome proliferator-activated receptor gamma (PPARgamma) activation of epididymally derived white adipocyte cultures reveals a population of thermogenically competent, UCP1-containing adipocytes molecularly distinct from classic brown adipocytes. The Journal of biological chemistry.

[43] Pisani DF, Djedaini M, Beranger GE, Elabd C, Scheideler M, Ailhaud G, Amri EZ (2011). Differentiation of Human Adipose-Derived Stem Cells into "Brite" (Brown-in-White) Adipocytes. Frontiers in endocrinology.

[44] Qiang L, Wang L, Kon N, Zhao W, Lee S, Zhang Y, Rosenbaum M, Zhao Y, Gu W, Farmer SR, Accili D (2012). Brown remodeling of white adipose tissue by SirT1-dependent deacetylation of Ppargamma. Cell.

[45] Betz MJ, Enerback S (2011). Therapeutic prospects of metabolically active brown adipose tissue in humans. Frontiers in endocrinology.

[46] Ravussin E, Galgani JE (2011). The implication of brown adipose tissue for humans. Annual review of nutrition.

[47] Vosselman MJ, van Marken Lichtenbelt WD, Schrauwen P (2013). Energy dissipation in brown adipose tissue: From mice to men. Molecular and cellular endocrinology.

[48] Wu J, Cohen P, Spiegelman BM (2013). Adaptive thermogenesis in adipocytes: is beige the new brown?. Genes & development.

[49] Liu Y, Zhang F, Zhang XF, Qi LS, Yang L, Guo H, Zhang N (2012). Expression of nucleophosmin/NPM1 correlates with migration and invasiveness of colon cancer cells. Journal of biomedical science.

[50] Mateen S, Raina K, Agarwal C, Chan D, Agarwal R (2013). Silibinin synergizes with histone deacetylase and DNA methyltransferase inhibitors in upregulating E-cadherin expression together with inhibition of migration and invasion of human non-small cell lung cancer cells. The Journal of pharmacology and experimental therapeutics.

[51] Dalerba P, Dylla SJ, Park IK, Liu R, Wang X, Cho RW, Hoey T, Gurney A, Huang EH, Simeone DM, Shelton AA, Parmiani G, Castelli C, Clarke MF (2007). Phenotypic characterization of human colorectal cancer stem cells. Proc Natl Acad Sci USA.

[52] Vermeulen L, Todaro M, de Sousa Mello F, Sprick MR, Kemper K, Perez Alea M, Richel DJ, Stassi G, Medema JP (2008). Single-cell cloning of colon cancer stem cells reveals a multi-lineage differentiation capacity. Proc Natl Acad Sci USA.

[53] Kumar S, Raina K, Agarwal C, Agarwal R (2014). Silibinin strongly inhibits the growth kinetics of colon cancer stem cell-enriched spheroids by modulating interleukin 4/6-mediated survival signals. Oncotarget.

[54] Ricci-Vitiani L, Lombardi DG, Pilozzi E, Biffoni M, Todaro M, Peschle C, De Maria R (2007). Identification and expansion of human colon-cancer-initiating cells. Nature.

[55] Fodde R, Brabletz T (2007). Wnt/beta-catenin signaling in cancer stemness and malignant behavior. Curr Opin Cell Biol.

[56] Onder TT, Gupta PB, Mani SA, Yang J, Lander ES, Weinberg RA (2008). Loss of E-cadherin promotes metastasis via multiple downstream transcriptional pathways. Cancer research.

[57] Thiery JP (2002). Epithelial-mesenchymal transitions in tumour progression. Nature reviews Cancer.

[58] Thompson EW, Newgreen DF, Tarin D (2005). Carcinoma invasion and metastasis: a role for epithelial-mesenchymal transition?. Cancer research.

[59] Ailles LE, Weissman IL (2007). Cancer stem cells in solid tumors. Curr Opin Biotechnol.

[60] Yao X, Shan S, Zhang Y, Ying H (2011). Recent progress in the study of brown adipose tissue. Cell & bioscience.

