# Supplementary material for: Functional modification of adipocytes by grape seed extract impairs their pro-tumorigenic signaling on colon cancer stem cells and the daughter cancer cells
Source: Oncotarget. 2014 Oct 10;5(20):10151–69. doi: 10.18632/oncotarget.2467 (PMC4259412; doi:10.18632/oncotarget.2467)
Supplement: Supplementary file 1 [file oncotarget-05-10151-s001.pdf]

SUPPLEMENTARY FIGURE AND TABLE

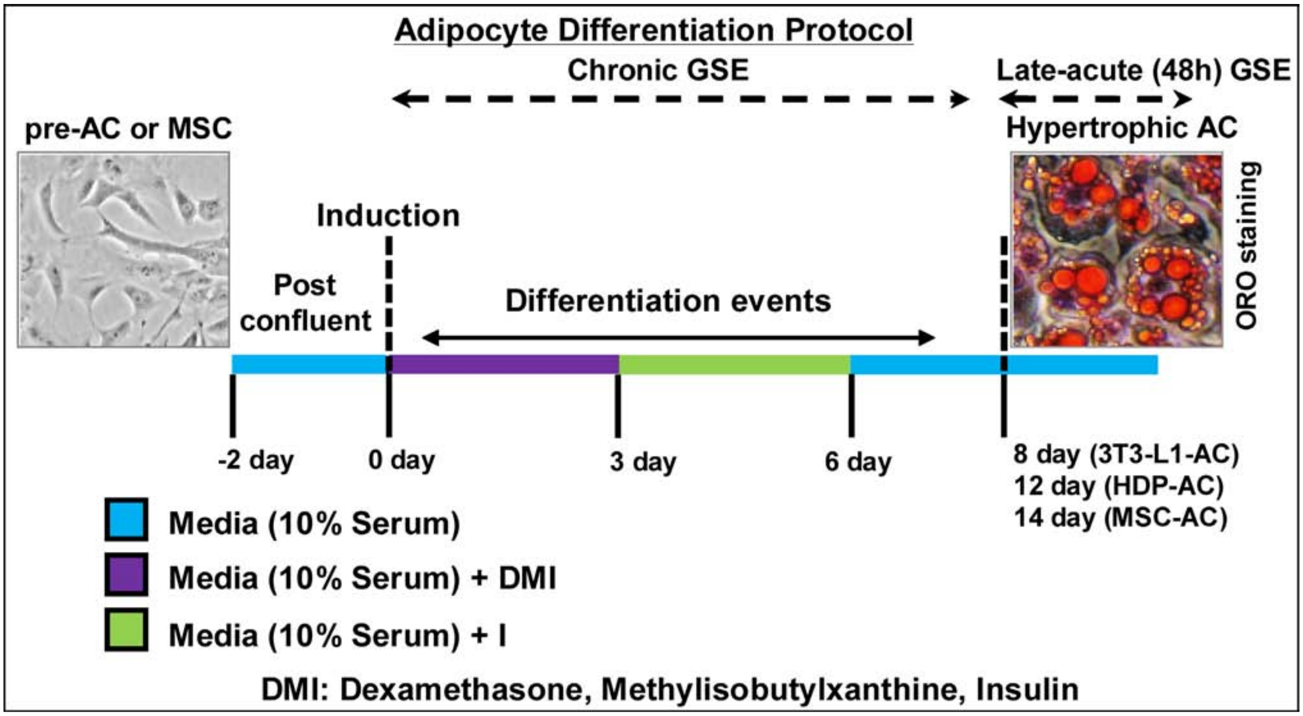

Supplementary Figure S1: Depiction of GSE treatment during different stages of adipocyte (AC) differentiation. Representative scheme for addition of additives during different stages of 3T3-L1-AC adipogenesis are shown. Phase contrast image on the left side of scheme represents the initial fibroblast/ pre-adipocyte or the mesenchymal stem cells before adipogenesis, while the ORO stained adipocytes on right side of scheme is a representative image of matured adipocytes. ORO stained red oil droplets help identify maturation of adipocytes; 3T3-L1-AC are matured by day 8 after induction of adipogenesis, while HDP-AC and MSC-AC are matured by day 12 and day 14, respectively. ORO: Oil Red-O stained lipids. Mouse 3T3-L1 differentiated adipocytes (3T3 L1-AC); human type II diabetic visceral adipocytes (HDP-AC); human adipocytes from adipose tissue derived mesenchymal stem cells (MSC-AC).

Supplementary Table S1. Primer sequence of genes used for semi-quantitative analysis by RT-PCR.

Gene	Primer (5'–3')
LGR5 (F)	GGTGACAACAGCAGTATGGACGA
LGR5 (R)	GAAGGTGAACACTGCACTGAATGAA
EpCAM (F)	AATGTGTGTGCGTGGGA
EpCAM (R)	TTCAAGATTGGTAAAGCCAGT
CD44 (F)	AGATCAGTCACAGACCTGCC
CD44 (R)	GCAAACTGCAAGAATCAAAGCC
CD133 (F)	CAGAGTACAACGCCAAACCA
CD133 (R)	AAATCACGATGAGGGTCAGC
OCT-4 (F)	TCCCATGCATTCAAACTGAGG
OCT-4 (R)	CCAAAAACCCTGGCACAAACT
NANOG (F)	TGGACACTGGCTGAATCCTTC
NANOG (R)	CGTTGATTAGGCTCCAACCAT
HES-1 (F)	TCAACACGACACCGGATAAA
HES-1 (R)	CCGCGAGCTATCTTTCTTCA
BMI-1 (F)	AATGTCTTTTCCGCCCGCT
BMI-1 (R)	ACCCTCCACAAAGCACACACAT
MSI-1 (F)	GAGGGTTCGGGTTTGTCACG
MSI-1 (R)	GGCGACATCACCTCCTTTGG
GAPDH (F)	CCCCTGGCCAAGGTCATCCA
GAPDH (R)	ACAGCCTTGGCAGCGCCAGT
